# A novel 4-gene signature for overall survival prediction in lung adenocarcinoma patients with lymph node metastasis

**DOI:** 10.1186/s12935-019-0822-1

**Published:** 2019-04-16

**Authors:** Yanfang Wang, Quanli Zhang, Zhaojia Gao, Shan Xin, Yanbo Zhao, Kai Zhang, Run Shi, Xuanwen Bao

**Affiliations:** 1 Ludwig-Maximilians-Universität München (LMU), 80539 Munich, Germany; 2 Jiangsu Key Laboratory of Molecular and Translational Cancer Research, Nanjing, 210009 China; 3 Department of Thoracic Surgery, The Affiliated Changzhou No. 2 People’s Hospital of Nanjing Medical University, Changzhou, 213000 China; 4 Institute of Molecular Toxicology and Pharmacology, Helmholtz Center Munich, German Research Center for Environmental Health, 85764 Neuherberg, Germany; 5 Department of Cardiology, Sir Run Run Shaw Hospital, Zhejiang University School of Medicine, Hangzhou, 310016 China; 6 Institute of Radiation Biology, Helmholtz Center Munich, German Research Center for Environmental Health, 85764 Neuherberg, Germany; 7 Technical University Munich (TUM), 80333 Munich, Germany

**Keywords:** Transcriptome, Lung adenocarcinoma (LUAD), Lymph node metastasis (LNM), mRNA signature, Weighted gene co-expression network analysis (WGCNA), Overall survival (OS)

## Abstract

**Background:**

Lung adenocarcinoma (LUAD) patients experiencing lymph node metastasis (LNM) always exhibit poor clinical outcomes. A biomarker or gene signature that could predict survival in these patients would have a substantial clinical impact, allowing for earlier detection of mortality risk and for individualized therapy.

**Methods:**

With the aim to identify a novel mRNA signature associated with overall survival, we analysed LUAD patients with LNM extracted from The Cancer Genome Atlas (TCGA). LASSO Cox regression was applied to build the prediction model. An external cohort was applied to validate the prediction model.

**Results:**

We identified a 4-gene signature that could effectively stratify a high-risk subset of these patients, and time-dependent receiver operating characteristic (tROC) analysis indicated that the signature had a powerful predictive ability. Gene Set Enrichment Analysis (GSEA) showed that the high-risk subset was mainly associated with important cancer-related hallmarks. Moreover, a predictive nomogram was established based on the signature integrated with clinicopathological features. Lastly, the signature was validated by an external cohort from Gene Expression Omnibus (GEO).

**Conclusion:**

In summary, we developed a robust mRNA signature as an independent factor to effectively classify LUAD patients with LNM into low- and high-risk groups, which might provide a basis for personalized treatments for these patients.

## Background

Lung cancer is the leading cause of cancer-related death worldwide, with adenocarcinoma being the most common histological type [1]. Despite advances in cancer therapy in recent decades, the prognosis of lung adenocarcinoma (LUAD) patients is still unfavourable, with an overall 5-year survival rate less than 15% [2]. The main reason for this low overall survival rate is that LUAD patients have a high frequency of lymph node metastasis (LNM) or even distant metastases at diagnosis [3–6]. Therefore, the identification of a high-risk subset from these patients who have greater need for additional systematic therapy is urgently needed.

In recent years, the development of gene expression profile technologies, such as microarray and next generation sequencing (NGS), have provided further opportunities to comprehensively characterize the molecular features of cancer [7, 8]. Considering that individual biomarkers usually have little statistical power, the current approach is to identify novel molecular signatures to offer better prediction in various cancers [9–11]. A number of studies have proposed gene expression-based signatures for survival stratification in patients with lung cancer [12–16]. However, few studies have focused on the prognostic prediction for LUAD patients with LNM.

In this study, based on The Cancer Genome Atlas (TCGA) LUAD mRNA-seq and clinical datasets, we sought to develop a gene expression signature to predict overall survival for LUAD patients with LNM, and then the proposed gene signature was validated in an external cohort from the Gene Expression Omnibus (GEO) database.

## Methods

### Data download and processing

TCGA RNA-seq datasets and clinical data for LUAD were downloaded by UCSC Xena browser (https://xenabrowser.net/). GSE68465 was download from the GEO database. The LUAD patients with LNM were filtered by the criteria that N stage of patients was I–IV.

### Co-expression gene network based on RNA-seq data

The Weighted Correlation Network Analysis (WGCNA) was used to construct the gene co-expression network [17]. The co-expression similarity $s_{i, j}$ was defined as the absolute value of the correlation coefficient between the profiles of nodes $i$ and $j$:

$$s_{i, j} = \left| {cor\left( {x_{i} , x_{j} } \right)} \right|$$

where $x_{i}$ and $x_{j}$ are expression values of for gene $i$ and $j$, and $s_{i, j}$ represent the Pearson’s correlation coefficients of gene $i$ and gene $j$, respectively.

A weighed network adjacency was defined by raising the co-expression similarity to a power $\beta$:

$$a_{i,j} = s_{i,j}^{\beta }$$

with $\beta$ ≥ 1 [18]. We selected the power of $\beta$ = 5 and scale free ${\text{R}}^{2}$ = 0.95 as the soft-thresholding parameters to ensure a signed scale-free co-expression gene network. Briefly, network construction, module detection, feature selection, calculations of topological properties, data simulation, and visualization were performed. Modules were identified via hierarchical clustering of the weighting coefficient matrix. The module membership of node $i$ in module $q$ can be defined as:

$$K_{cor,i}^{\left( q \right)} : = cor\left( {x_{i} ,E^{\left( q \right)} } \right)$$

where $x_{i}$ is the profile of node $i$, and ${\text{E}}\left( q \right)$ is the module eigengene (the first principal component of a given module) of module $q$ [19]. The module membership measure $K_{cor,i}^{\left( q \right)}$, lies in $\left[ { - 1, 1} \right]$ and specifies how close node $i$ is to module $q$, $q = 1, \ldots , Q$.

The topological overlap measure (TOM) plots visualized inter-connectivity patterns and suggests the presence of large modules. This property of dense connections among the genes of module $q$ can be measured using the concept of module density, which is defined as the average adjacency of the module genes:

$$Density\left( {A^{\left( q \right)} } \right) = \frac{{\mathop \sum \nolimits_{i} \mathop \sum \nolimits_{j \ne i} a_{i,j}^{\left( q \right)} }}{{n^{\left( q \right)} \left( {n^{\left( q \right)} - 1} \right)}}$$

where $A^{\left( q \right)}$ denotes the $n^{\left( q \right)}$ × $n^{\left( q \right)}$ adjacency matrix corresponding to the sub-network formed by the genes of module $q$. By evaluating the correlations between the LNM status of LUAD and their module memberships, highly correlated modules can be identified. The modules that had correlation coefficient with N status larger than 0.15 or less than − 0.15 were selected.

### Differentially expressed gene (DEG) analysis

DEG analysis was performed by the Limma package [20]. The tissue samples were separated into para-tumour group and tumour group. The DEGs were defined as genes with Q value (adjusted p value between two groups) less than 0.05.

### Cox regression

Cox regression, also called Proportional Hazards Regression, is a survival analysis model [21]. It can be used to analyse relationships between different features and the survival time. The Cox model is based on the proportional hazards condition, which assumes that features have a proportional relationship to the exponential change of a hazard. Thus, the model is formulated as a multiplication of a baseline hazard function with a sole time variable, $t$, and an exponential function of the linear combination of all of the features as an input. Given a set of $n$ samples $\left\{ {\left( {\varvec{X}_{i} ,Y_{i} ,s_{i} } \right) | 0 \le i \le n, i \in \varvec{R}} \right\}$, where $X_{i} = \left( {x_{i0} ,x_{i1} , \ldots ,x_{ik} } \right)$ and stands for the $i$-th sample of all the $k$ features, $Y_{i}$ is the observation time and $s_{i}$ is the survival status, the hazard function is

$$H_{i} \left( t \right) = H_{0} \left( t \right)e^{{\varvec{X}_{\varvec{i}}^{T}\varvec{\beta}}}$$

$\varvec{\beta}= \left( {\beta_{0} ,\beta_{1} , \ldots ,\beta_{k} } \right)$ is the coefficient vector weighing the contribution of the features. The partial likelihood of all the samples is

$$\begin{aligned} L\left(\varvec{\beta}\right) = & \mathop \prod \limits_{i = 1}^{n} L_{i} \left(\varvec{\beta}\right) \\ = & \mathop \prod \limits_{i = 1}^{n} \frac{{H_{i} \left( {Y_{i} | \varvec{X}_{i} } \right)}}{{\mathop \sum \nolimits_{{j:Y_{j} \ge Y_{i} }} H_{i} \left( {Y_{i} | \varvec{X}_{j} } \right)}} \\ = & \mathop \prod \limits_{i = 1}^{n} \frac{{e^{{\varvec{X}_{\varvec{i}}^{T}\varvec{\beta}}} }}{{\mathop \sum \nolimits_{{j:Y_{j} \ge Y_{i} }} e^{{\varvec{X}_{\varvec{j}}^{T}\varvec{\beta}}} }} \\ \end{aligned}$$

By penalizing −log ($L\left(\varvec{\beta}\right)$), the optimal $\varvec{\beta}$ can be uncovered.

### LASSO regularization

LASSO (Least Absolute Shrinkage and Selection Operator) is an important regularization in many regression analysis methods (e.g., COX regression, logistic regression) [22]. The idea behind LASSO is that an L1-norm is used to penalize the weight of the model parameters. Assuming a model has a set of parameters $\left\{ {w_{0} , w_{1} , \ldots , w_{n} } \right\}$, the LASSO regularization can be defined as

$$\lambda \cdot \mathop \sum \limits_{i = 0}^{n} \left\| {\left. {w_{i} } \right\|} \right._{1}$$

It can also be expressed as a constraint to the targeted objective function

$$\sum \left\| \varvec{Y} \right. - \left. {\varvec{Y}^{*} } \right\|_{2} ,\quad s.t. \left\| {\left. {w_{i} } \right\|} \right._{1} < t$$

An important property of the LASSO regularization term is that it can force the parameter values to be 0, thus generating a sparse parameter space, which is a desirable characteristic for feature selection. In our analysis, the overlapping genes from DEGs and selected modules were used as the input of Lasso Cox regression. The nomogram was done by rms package [23]. The GSEA was done by GSEA software from Broad institute [24].

## Results

**Fig. 1:**
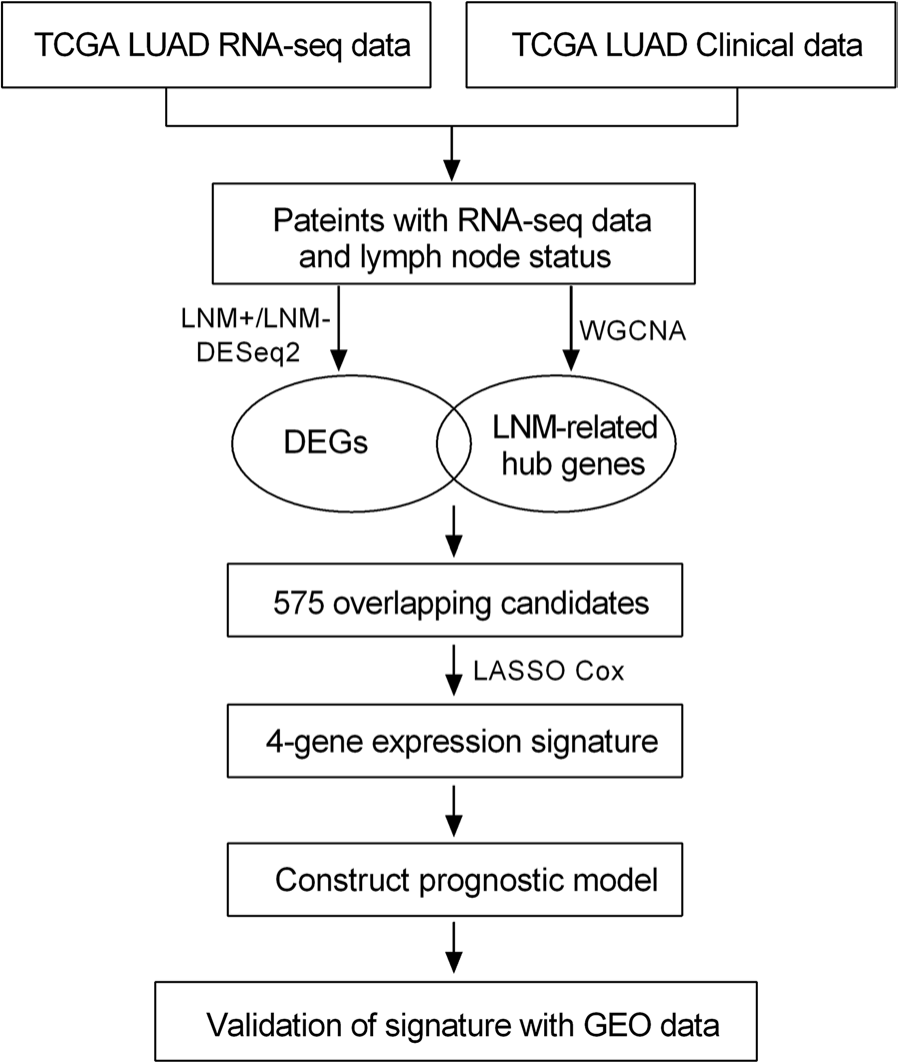
Flowchart of this study

The flowchart of our study is shown in Fig. 1. By integrating the TCGA LUAD mRNA-seq dataset with the clinical dataset, 575 promising candidates were filtered out and submitted for LASSO Cox regression analysis to identify robust markers to construct a prognostic signature. Then, a GEO dataset (GSE68465) was used to validate the model.

### Identification and selection of prognostic biomarkers in LNM-positive patients

**Fig. 2:**
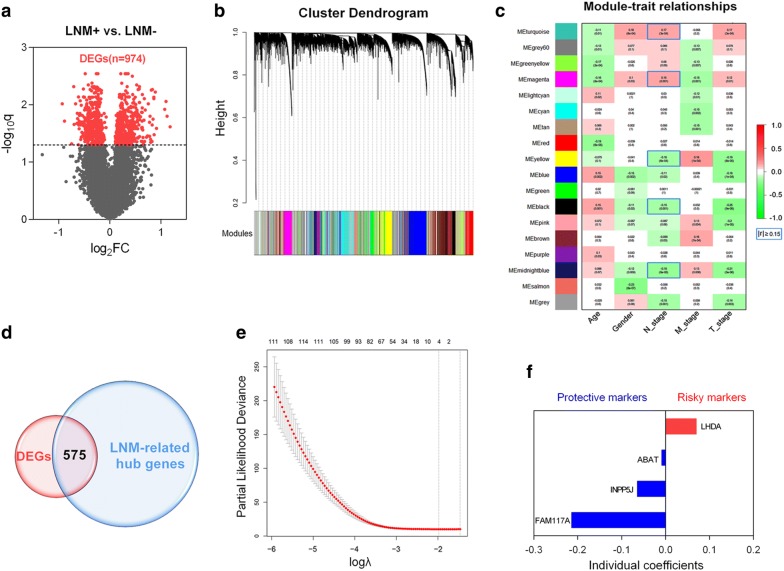
Identification of prognostic genes in LNM-positive patients. **a** Volcano plot showing DEGs in LNM + samples. **b** Clustering dendrogram of genome-wide genes in LNM + samples. **c** Correlation between modules and traits. Absolute values of correlation coefficients between LNM-status and modules greater than 0.15 were considered as LNM-related modules. **d** Five hundred seventy-five overlapping candidates in the intersection of two sets. **e** LASSO Cox analysis identified 4 genes most correlated with overall survival in the training set. **f** Cox coefficients distribution of the gene signature

The whole transcriptome of LNM-positive samples was included to perform DEG analysis, and the volcano plot showed 974 differentially expressed genes (DEGs) in 173 LNM-positive (LNM+) tumour samples compared to 335 LNM-negative (LNM-) tumour samples (Fig. 2a). To construct gene co-expression modules, RNA-seq data from the whole genome was subjected to WGCNA. Genes were assigned to different modules by cluster dendrogram trees, and unassigned genes were categorized into the grey module (Fig. 2b). The relationships between clinical traits and gene modules are presented in Fig. 2c. Absolute values of correlation coefficients between LNM status and modules greater than 0.15 were considered as LNM-related modules, and genes in these modules were extracted for further study. As shown in Fig. 2d, we obtained 575 overlapping genes in the intersection of DEGs and LNM-related hub genes.

Then, LASSO Cox regression analysis was performed to identify robust markers among the 575 candidates. By forcing the sum of the absolute value of the regression coefficients to be less than a fixed value, certain coefficients were shrunk to exactly zero, and the most powerful prognostic markers were identified with relative regression coefficients. Cross-validation was applied to prevent the over-fitting of the LASSO Cox model (Fig. 2e). Figure 2f shows individual coefficient distributions of the 4 filtered markers: LDHA was associated with high risk (HR > 1), while ABAT, INPP5J and FAM117A were shown to be protective (HR < 1).

### Risk score and survival prediction based on the 4-gene signature

**Fig. 3:**
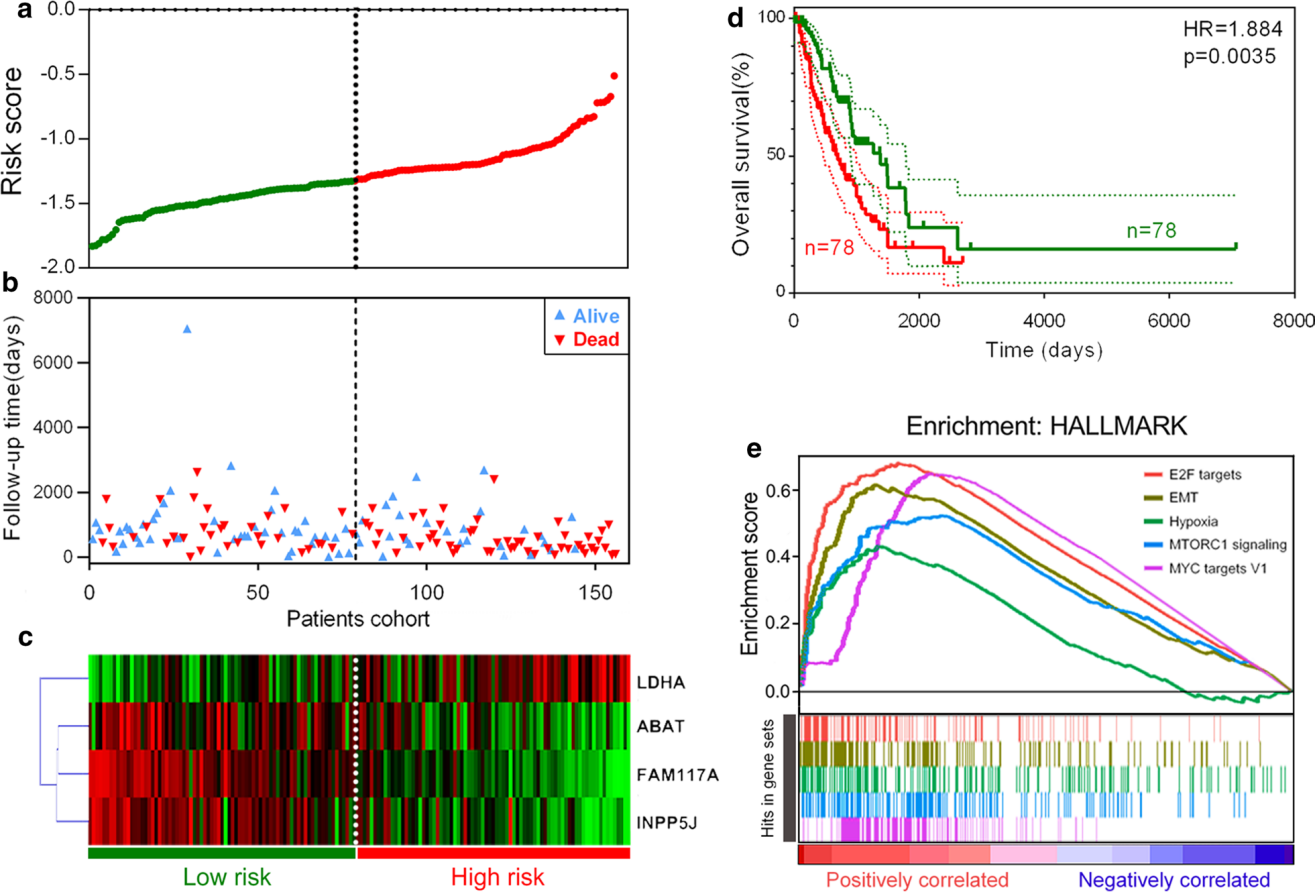
Signature-based risk score is a promising marker in the training cohort. **a** Risk score distribution. **b** Survival overview. **c** Heatmap showing the expression profiles of the signature in low- and high-risk groups. **d** Patients in the high-risk group exhibited worse overall survival compared to those in the low-risk group. **e** GSEA revealed most significant hallmarks correlated with the high-risk group

To comprehensively investigate the association between the 4 identified genes and prognosis in these patients, we developed a 4-gene signature-based prognostic model according to their individual coefficients. Then, we calculated the risk score for each LNM+ patient in the training set and ranked them. Thus, LNM+ patients with follow-up information were divided into two groups: a low-risk group (n = 78) and a high-risk group (n = 78) based on median cut-off value (Fig. 3a). Figure 3b shows the survival overview in the training cohort. A heatmap showed that patients in the high-risk group have a tendency to have higher expression of LDHA and lower expression of ABAT, FAM117A and INPP5J (Fig. 3c). The Kaplan–Meier curve and log-rank test suggested that patients in the high-risk group have significantly worse overall survival compared to those in the low-risk group (HR = 1.884, p = 0.0035) (Fig. 3d). As shown in Fig. 3e, GSEA showed the top 5 hallmarks correlated with the high-risk group: E2F targets, EMT, Hypoxia, MTORC1 signalling and MYC targets (FDR q < 0.001).

### Expression profiles of the 4-gene signature and subgroup analysis

**Fig. 4:**
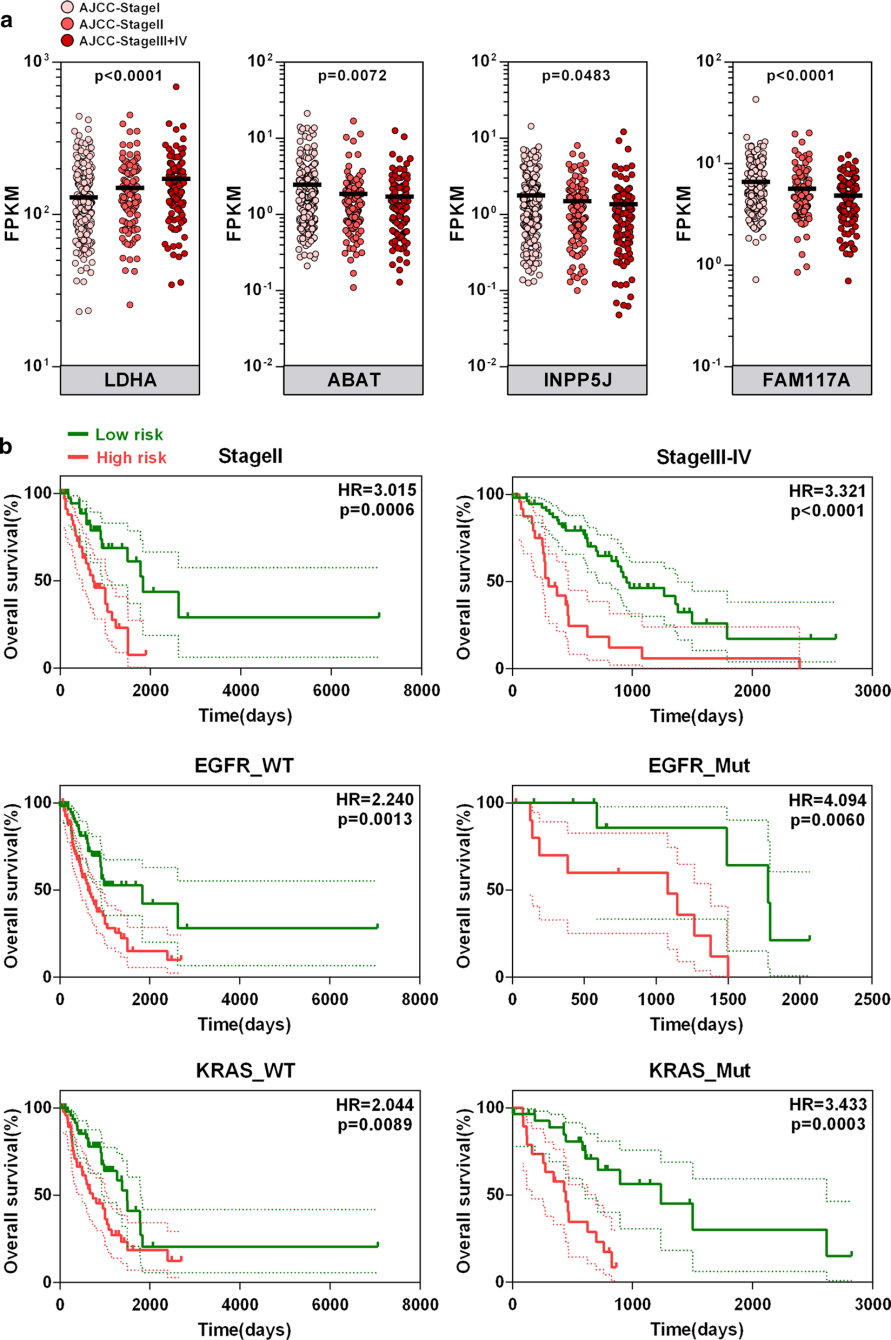
Expression and survival analysis in subgroups. **a** Expression pattern of the gene signature in different AJCC-TNM stages. **b** Signature-based risk score is a promising marker for overall survival in subgroups with different tumour stages and EGFR and KRAS statuses

We investigated the 4 genes’ expression profiles in different AJCC-TNM stages in the TCGA cohort. As shown in Fig. 4a, one-way ANOVA test showed that LDHA mRNA expression was significantly upregulated, while ABAT, FAM117A and INPP5J were significantly downregulated, in more advanced stages. As shown in Fig. 4b, the signature-based risk score also serves as a promising marker to predict overall survival in different subgroups, including stage II (HR = 3.015, p = 0.0006), stage III-IV (HR = 3.321, p < 0.0001), EGFR-wild-type (EGFR-WT) (HR = 2.240, p = 0.0013), EGFR-mutated (EGFR-Mut) (HR = 4.094, p = 0.0060), KRAS-wild type (KRAS-WT) (HR = 2.044, p = 0.0089) and KRAS-mutated (KRAS-Mut) (HR = 3.433, p = 0.0003) patients, respectively.

### Construction of a nomogram to predict overall survival in LNM-positive patients

**Fig. 5:**
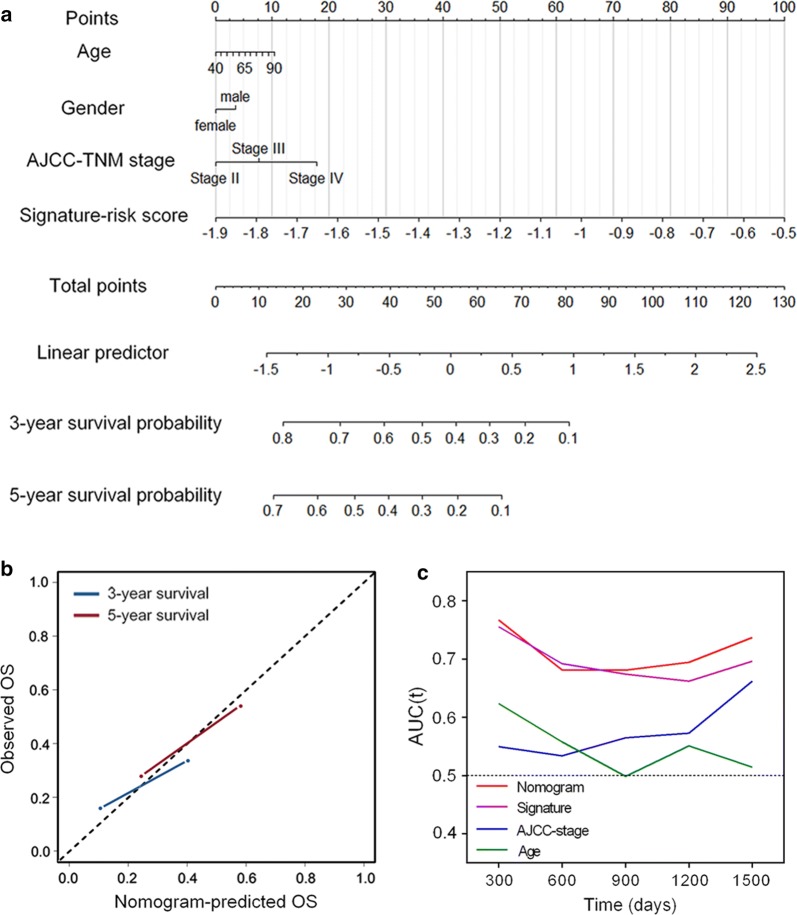
Construction of a nomogram for survival prediction. **a** Nomogram combining signature with clinicopathological features. **b** Calibration plot showing that nomogram-predicted survival probabilities corresponded closely to the actual observed proportions. **c** The AUC(t) of multivariable models indicated the nomogram had the highest predictive power for overall survival

Then, we constructed a nomogram that integrated clinicopathological features with the 4-gene signature to predict survival probability of LNM + patients (Fig. 5a). Calibration plot showed that the nomogram-predicted 3-year and 5-year survival probabilities and corresponded closely to the actual observed proportions (Fig. 5b). The AUC(t) functions of the multivariable models were developed to indicate how well these features serve as prognostic markers. Compared to other features, such as signature-based risk score, AJCC-TNM stage and age, the nomogram showed the highest predictive power for overall survival in the training cohort, with an average AUC above 0.7 in the follow-up period (Fig. 5c).

### Validation of the 4-gene signature for survival prediction

**Fig. 6:**
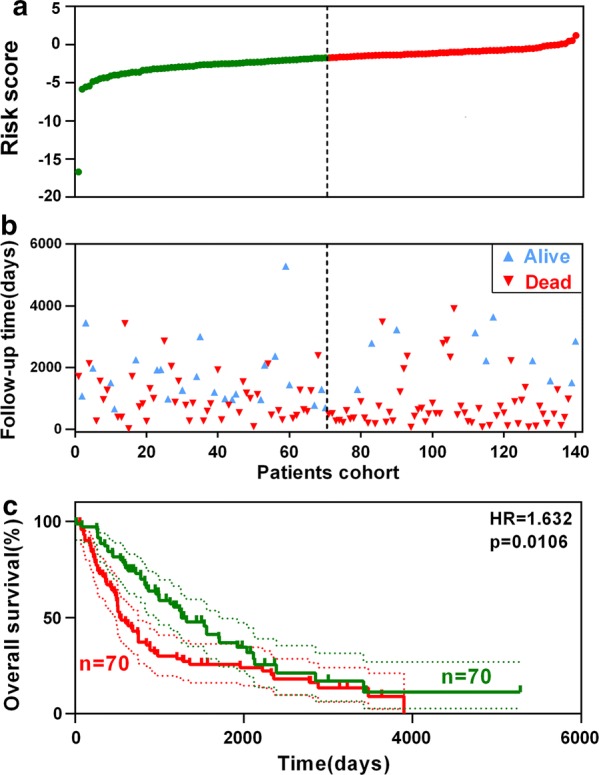
Validation of the signature in an external cohort. **a** Risk score distribution. **b** Survival overview. **c** Patients with a high risk score exhibited poorer overall survival in the validation cohort

To confirm our findings in the training set, we validated the prognostic function of the 4-gene signature in an independent GEO cohort (GSE68465). After extracting the microarray data of 140 LNM+ patients with follow-up information, we calculated the risk score for each patient by using the same formula in the training set. Figure 6a shows the risk score distribution, and Fig. 6b shows the survival overview in the validation cohort. According to the median cut-off value, the cohort of patients were divided into high- (n = 70) and low-risk (n = 70) groups. The Kaplan–Meier curve suggested a significant better overall survival in the low-risk group compared to the high-risk group (HR = 1.632, p = 0.0106) (Fig. 6c). The result was consistent with our previous findings in the training cohort based on TCGA dataset, indicating the gene signature was validated as a reliable predictor for overall survival in an independent LNM + LUAD patient cohort.

## Discussion

Accumulating evidence shows that lung adenocarcinoma patients with local invasion or lymph node metastasis always exhibit poor responses to standard treatments and thus tend to have poor clinical outcomes [3–5, 25]. In clinical practice, these patients need more intensive monitoring and aggressive therapy, and robust biomarkers are urgently needed to stratify high-risk groups among these patients. However, individual biomarkers usually have very little predictive power. The established clinical markers for survival are primarily based on patient- and tumour-related factors, such as AJCC-TNM stage, while the accuracy and specificity are also limited. Therefore, our study aimed to identify novel molecular signatures integrated with established clinicopathological features to predict overall survival in LUAD patients with lymph node metastasis.

In this study, we identified 4 coding genes associated with overall survival in LUAD patients with lymph node metastasis, namely, LDHA, ABAT, FAM117A and INPP5J, in the training set. Among the 4 coding genes, LDHA was widely reported to promote malignant progress and predict poor survival in various cancer types [26–30]. In addition, Ooms et al. reported that INPP5 J functions as a tumour suppressor to inhibit breast cancer cells’ invasive ability via PI3 K/AKT signalling [31]. However, ABAT and FAM117A remain inadequately investigated in cancer-related research. To some degree, our study might provide some clues for further investigation on the biological roles and clinical significance of these genes.

Based on multivariate Cox coefficients derived from LASSO analysis, we developed a 4-gene signature-based risk score model. Moreover, we investigated the prognostic value of the signature in different subgroups. In detail, the signature still exhibited powerful prediction for overall survival in LNM+ patients with same TNM stage and genomic alteration (including EGFR and KRAS mutation status), confirming that the signature is a promising marker independent of different clinicopathological features. In addition to survival prediction, GSEA showed that the signature-identified high-risk group was significantly correlated with certain hallmarks of cancer, such as EMT and hypoxia, indicating the potential molecular mechanisms underlying the lethal tendency of these LNM+ patients. By integrating established clinicopathological features with the signature, we developed a nomogram to predict the survival probability of LNM+ patients. The predictive power was measured by the time-dependent area under the ROC curve (AUC(t)), and the result showed that the integrated nomogram model had higher predictive power than individual markers. Lastly, an external GEO cohort was used to validate the prognostic value of the 4-gene signature, and the survival analysis showed the same tendency in the validation cohort.

The limitations of our study should be acknowledged. This is a retrospectively designed study, and the sample size of the training and validation sets is relatively small.

In summary, the novel 4-gene signature proved to be a robust model with high predictive power in LUAD patients with LNM+. The predictive power was stable over time and showed promising survival prediction in combination with established markers. The use of the signature integrated with clinicopathological features can help to further stratify LNM+ patients into risk groups, thus serving as a predictive tool for clinical outcome, guiding personalized treatment, and resulting in more aggressive therapy for high-risk patients or less aggressive therapy for low-risk patients. Further research is needed to reveal the interplay between these genes, and thereby, we will be able to develop better treatment alternatives for high-risk LUAD patients with lymph node metastasis.

## Conclusion

In conclusion, based on publicly available data, we constructed a robust mRNA signature that could serve as a reliable marker to stratify a high-risk group among LUAD LNM+ patients. Subgroup analysis indicated that the signature works effectively independent of other clinical features. Validation in an external cohort from GEO further confirmed the prognostic value of the signature. We hope the identified signature could help to improve the strategies for personalized treatment of LUAD patients with LNM.

## Abbreviations

LUAD: lung adenocarcinoma
LNM: lymph node metastasis
TCGA: The Cancer Genome Atlas
tROC: time-dependent receiver operating characteristic
GSEA: Gene Set Enrichment Analysis
GEO: Gene Expression Omnibus
WGCNA: weighted gene co-expression network analysis
OS: overall survival
NGS: next generation sequencing
DEG: differentially expressed gene
LASSO: Least Absolute Shrinkage and Selection Operator
